# Review of the aetiologies of central nervous system infections in Vietnam

**DOI:** 10.3389/fpubh.2024.1396915

**Published:** 2025-01-31

**Authors:** Hannah E. Brindle, Marc Choisy, Robert Christley, Neil French, Michael Griffiths, Pham Quang Thai, H. Rogier van Doorn, Behzad Nadjm

**Affiliations:** ^1^Institute of Infection, Veterinary and Ecological Sciences, Faculty of Health and Life Sciences, University of Liverpool, Liverpool, United Kingdom; ^2^Oxford University Clinical Research Unit, Hanoi, Vietnam; ^3^Oxford University Clinical Research Unit, Ho Chi Minh City, Vietnam; ^4^Centre for Tropical Medicine and Global Health, University of Oxford, Oxford, United Kingdom; ^5^National Institute of Hygiene and Epidemiology, Hanoi, Vietnam; ^6^The Medical Research Council, The Gambia at London School of Hygiene and Tropical Medicine, Fajara, Gambia

**Keywords:** central nervous system infection (CNSi), Vietnam, Japanese encephalitis, *Streptococcus suis*, meningitis, encephalitis

## Abstract

Central nervous system (CNS) infections are an important cause of morbidity and mortality in Vietnam, with many studies conducted to determine the aetiology. However, the cause remains unknown in a large proportion of cases. Although a systematic review of the aetiologies of CNS infections was conducted in the Mekong region, there are no known published reviews of the studies specifically in Vietnam. Here, we review the cause of CNS infections in Vietnam while also considering the potential aetiologies where a cause was not identified, based on the literature from the region. In particular, we focus on the most common pathogens in adults and children including *Streptococcus suis* which is associated with the consumption of raw pig products, and Japanese encephalitis virus, a mosquito-borne pathogen. We also discuss pathogens less commonly known to cause CNS infections in Vietnam but have been detected in neighbouring countries such as *Orientia tsutsugamushi*, *Rickettsia typhi* and *Leptospira* species and how these may contribute to the unknown causes in Vietnam. We anticipate that this review may help guide future public health measures to reduce the burden of known pathogens and broaden testing to help identify additional aetiologies.

## Introduction

Central nervous system (CNS) infections are an important cause of morbidity and mortality worldwide with the highest burden in low and middle-income countries. In 2016, encephalitis resulted in 103,000 deaths and 670.4 million Disability-Adjusted Life Years (DALYS) and meningitis, 318,000 deaths and 21.9 million DALYS (1). Vietnam is located in Southeast Asia, neighbouring or in proximity to Cambodia, Laos, Thailand, Myanmar and China. This subregion is a ‘hotspot’ for emerging and zoonotic infections due to increasing urbanisation, cross-border population movements, farming practices and animal trade, and climate change (2).

In a systematic review of the aetiologies of encephalitis in the Mekong region (defined as Cambodia, Laos, Vietnam, Thailand and Yunnan province in China), Japanese encephalitis virus (JEV) was the most commonly referenced pathogen, accounting for 47% (n = 35) of cases (3). However, there are no known published reviews of the studies conducted only in Vietnam.

This review will describe the prevalence, the epidemiology and the risk factors of the common causes of encephalitis and meningitis in Vietnam based on publications since 1998 (Supplementary Tables S1, S2) (4–15). It will also discuss the rarer causes of CNS infections in the wider region which may be contributing to cases of unknown aetiology in the country.

## Bacteria

### Streptococcus suis

*Streptococcus suis*, a Gram-positive coccus, was first detected in Vietnam in 1997 (16, 17) and became a notifiable disease in 2011 (18). The pathogen is found in the gastro-intestinal, genital and respiratory tract of pigs who can develop complications including encephalitis and arthritis (19, 20). Human become infected after consuming or being exposed via skin abrasions, to undercooked pig blood or products (21–23). The incidence of *S. suis* in humans is highest in Southeast Asia where contact with pig products is more common (24). *S. suis* was first detected in Vietnam in 1997 and became a notifiable disease in 2011 (18). The most recent available national surveillance data are from 2017 when there were 178 reported cases (25).

**Figure 1:**
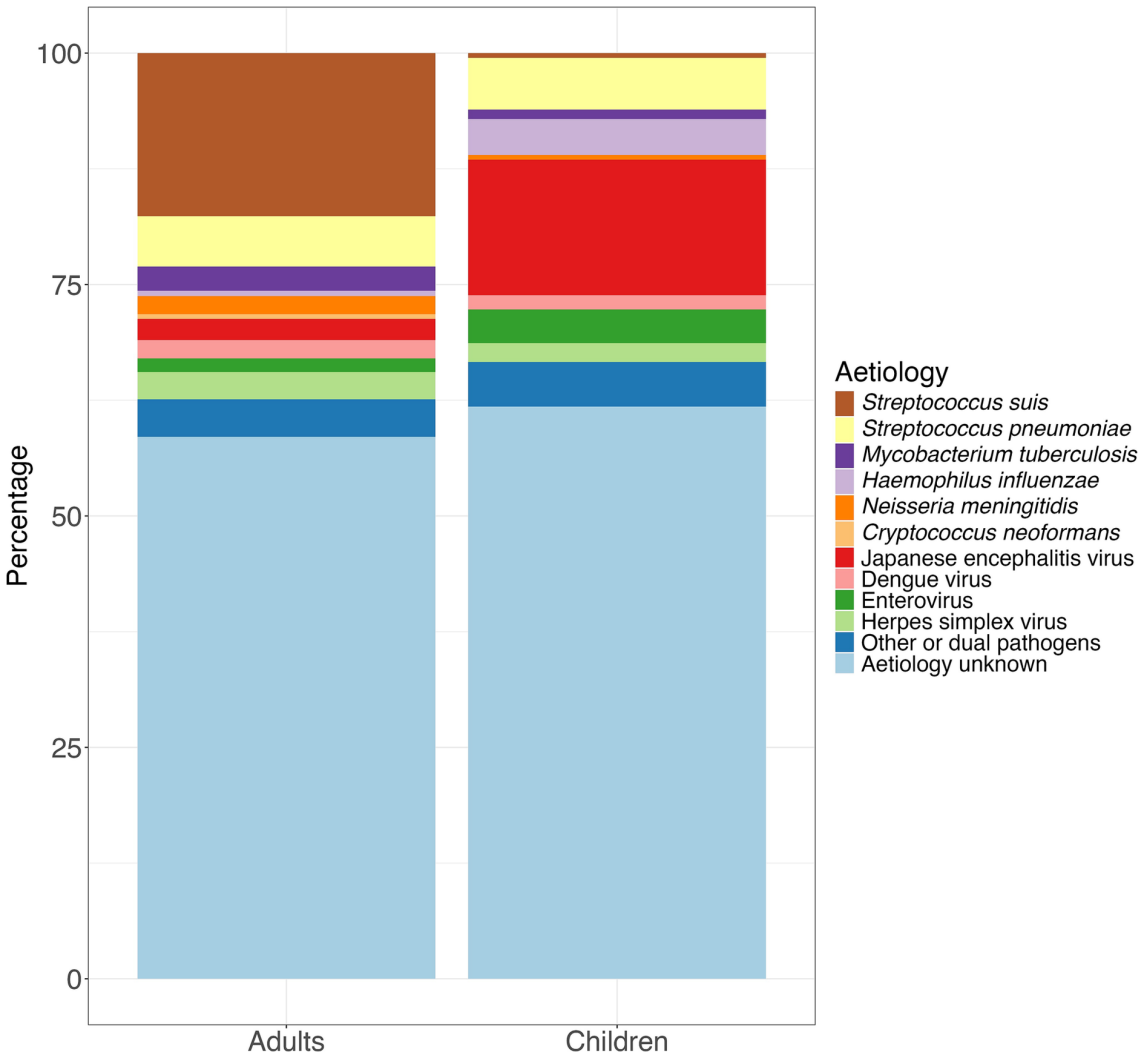
Percentage of aetiologies among adults and children based on the total number of pathogens detected in the 12 studies included in Supplementary Table S1.

Male gender, older age, having diabetes mellitus and excessive alcohol consumption are all associated with increased risk of infection (21, 26, 27) and, although it can cause septicaemia, the most common presentation is meningitis (17). Infections in children are very rare as they as less likely to have contact with the raw or uncooked pig products (21). In northern Vietnam, the incidence of human *S. suis* was shown to be correlated with pig density (26) and it has been suggested that infections of *S. suis* in humans may correspond with outbreaks of Porcine Respiratory and Reproductive Syndrome (PRRS) which is associated with secondary bacterial infections in pigs (23, 26). No vaccines currently exist against *S. suis* for humans although candidates are in the pipeline (28). While there are no commercial porcine vaccines, autologous vaccines are used in some circumstances (29). Of the studies outlined in Supplementary Table S1, *S. suis* was the most common pathogen in adults accounting for 17.6% (*n* = 448) of pathogens. However, it is much less common in children accounting for only 0.5% (*n* = 10) of pathogens (Supplementary Tables S3, S4; Figure 1).

In a study of 450 patients admitted to the Hospital for Tropical Diseases in Ho Chi Minh City between 1997 and 2004 with suspected bacterial meningitis, the most common symptoms were headache and neck stiffness, both accounting for 94% (*n* = 142) of the cases, followed by vomiting which accounted for 66.2% (*n* = 100) of the cases. Forty-seven (31.1%) of the cases had a Glasgow Coma Score of less than 11. While only four cases died, 40 cases still had sequelae at 6 months after discharge with 14 suffering from severe hearing loss (30). In a more recent study of the outcomes of patients with *S. suis* admitted to the National Hospital for Tropical Diseases in Hanoi between November 2014 and October 2015, of whom 97.4% (*n* = 76) had meningitis, the prevalence ratio of vestibular dysfunction at 9 months after discharge in those recruited prospectively (*n* = 47) compared to controls was 1.8 (95%CI 1.1–2.5) compared to controls (31).

### Streptococcus pneumoniae

*Streptococcus pneumoniae*, a Gram-positive coccus, colonises the upper respiratory tract and is transmitted between humans via contact with infected secretions (32). Children, older people and those with immunosuppression are at greater risk of developing invasive disease such as pneumonia, meningitis and sepsis (33). In many countries*, S. pneumoniae* is the most common cause of bacterial meningitis (34). In Vietnam, it was reported as the second most common pathogen causing CNS infection in both adults (5.4%, *n* = 138) and children (5.6%, *n* = 109); (Supplementary Tables S3, S4; Figure 1). In some countries in the global north, where the pneumococcal vaccine has been rolled-out, a reduction in the incidence of pneumococcal meningitis has been seen (34). The pneumococcal vaccine has not yet been introduced in Vietnam, although studies have been conducted to help determine the method of introduction and dosing schedules (35, 36) and it is due to be included in the Expanded Programme for Immunisation (EPI) in 2025.

### Mycobacterium tuberculosis

The intracellular mycobacterium *Mycobacterium tuberculosis* (MTB) is transmitted via aerosolised droplets and can result in pulmonary or less, commonly, extrapulmonary disease (37). It is estimated that 1% of infections result in tuberculous meningitis (TBM) (38) and in a study conducted in Ho Chi Minh City between 2009 and 2011, the majority of cases of TBM occurred in young children (39). Although data from Vietnam in 2022 showed that coverage of the Bacillus Calmette-Guérin (BCG) among those aged one-year in Vietnam was 88% (43), it is possible that TBM remains under-diagnosed including in recent studies where the percentage of cases was less than 3% in both adults and children (Figure 1; Supplementary Tables S3, S4). Laboratory diagnosis of TBM is challenging. A separate study conducted in Ho Chi Minh City (HCMC), Vietnam suggested that at least 6 mL of cerebrospinal fluid (CSF) is needed to culture *M. tuberculosis* and microscopy using the Ziehl-Neelsen (ZN) method needs to be performed for at least 30 minutes to identify acid-fast bacilli (40). GeneXpert MTB/RIF Ultra is now recommended by the World Health Organisation as the first line diagnostic test for TBM although this had lower negative predictive value in studies where a higher proportion of cases were human immunodeficiency virus (HIV) negative, including in Vietnam (41, 42). Given the challenges with differentiating between bacterial meningitis and TBM solely on clinical features, a diagnostic aid was developed based on age, duration of illness and laboratory features from 251 adults admitted to the Clinical Research Unit at the Centre for Tropical Diseases in Ho Chi Minh City between 1997 and 2000. This tool had a sensitivity of 86% and a specificity of 79% when applied prospectively to 75 adults with either tuberculous or bacterial meningitis (44).

The prognosis for TBM remains poor. In a study conducted in Ho Chi Minh City, 15% (*n* = 15) of children with TBM died and 33% (*n* = 27) had a disability at the end of treatment (39). Much research has been conducted in Vietnam on the use of corticosteroids for the treatment of TBM. A randomised, double-blind controlled trial of 545 patients over 14 years of age with TBM conducted in Ho Chi Minh City, showed that the use of dexamethasone reduced the risk of death compared to a placebo (relative risk 0.69; 95% confidence interval (CI) 0.52–0.92, *p* = 0.01) (45). However, a separate trial also conducted in Ho Chi Minh City found that there was no benefit using dexamethasone compared to placebo among HIV positive adults (46).

### Haemophilus influenzae

*Haemophilus influenzae*, is a Gram negative coccobacillus which can colonise the nasopharynx, especially in children, and can be transmitted via nasal secretions (47, 48). In some cases, it can cause a localised infection such as sinusitis or otitis media and less commonly, pneumonia and meningitis. Invasive disease is mainly due to capsular type b (Hib). The expansion of the vaccine is likely to have contributed to the global decline in cases of meningitis due to Hib from 435,000 cases in 1990 to 187,000 cases in 2019 (a 56.9% reduction), and deaths from 38,000 in 1990 to 11,100 (a 70.8% reduction) (49). In a study conducted in Ho Chi Minh City between 1993 and 1996, cases of Hib accounted for 35% of cases of meningitis in children (4). However, the Hib conjugate vaccine was introduced into the Vietnam National Expanded Programme on Immunisation (EPI) in June 2010 (9) and as a result, cases of Hib cases in children decreased from 34.9% (*n* = 30) in a study starting in 1995 (4) to 0.5% (*n* = 1/203) in a study starting 2014 (Figure 1; Supplementary Tables S3, S4) (14).

### Neisseria meningitidis

*Neisseria meningitidis*, a Gram negative coccus, can colonise the nasopharynx and is transmitted via aerosol or secretions (50, 51). Invasive disease can manifest as meningitis, septicaemia, or both (52). In southern Vietnam, the incidence rate between 2011 and 2021 was 0.02 per 100,000 persons/year with serogroup B accounting for 90% of cases (53). However, *N. meningitidis* it is one of the less common pathogens reported in Vietnam accounting for less than 2% of pathogens in both adults and children (Figure 1; Supplementary Tables S3, S4) (21–23). A recent study showed that antimicrobial resistance, most frequently to chloramphenicol and ciprofloxacin, was common among isolates of invasive meningococcal disease (53). As the availability of meningococcal vaccines increased globally, due to the increase in availability of the meningococcal vaccines, global case numbers of *N. meningitidis* decreased from 744,000 in 1990 to 433,000 in 2019 (a 41.7% reduction) and deaths from 80,900 to 32,100 (a 60.2% reduction) (49) Vaccination in Vietnam is only available for groups at high-risk and is not part of the national immunisation programme (54). However, in 2022, an expert advisory group recommended that the four component meningococcal B vaccine (4CMenB) start as soon as possible in children (55).

### *Orientia tsutsugamushi* (scrub typhus)

The Gram-negative obligate intracellular bacterium *Orientia tsutsugamushi,* the cause of scrub typhus, is transmitted by the bite of an infected *Leptotrombidium delicense* (trombiculid) mite or ‘chigger’ which is found in damp soil or detritus in wild, overgrown areas (56–59). A study conducted in Hanoi found that 55.7% (*n* = 140) of the patients admitted to hospital with scrub typhus from 2001 to 2003 were farmers (60). Those with scrub typhus develop an eschar, an area of skin necrosis at the site of the bite and localised lymphadenopathy. Clinical symptoms of scrub typhus may then range from fever, cough, headache and myalgia to meningitis and/or multi-organ failure in severe cases (56, 61, 62).

The diagnosis of scrub typhus is challenging as *Orientia tsutsugamushi* is not easily cultured, the PCR detection of DNA in blood has a low sensitivity, and the gold standard indirect immunofluorescence using paired sera (60) is rarely available and requires high levels of expertise. It is therefore possible, that cases of CNS infection due to *Orientia tsutsugamushi* are under-reported, particularly in adults. In recent studies of the aetiology of CNS infections in Vietnam, only one case was detected (in a child) (14). Despite this, in a study of patients of all ages with altered consciousness or neurological findings in Laos, 2.9% (*n* = 31) had *O. tsutsugamushi* (63) and in a study of patients aged two months to 78 years with acute encephalitis syndrome (AES) in Lucknow, India, 8.7% (*n* = 357) were diagnosed with the pathogen (64).

### Rickettsia typhi

Murine typhus caused by *Rickettsia typhi*, is transmitted when the faeces of infected fleas, which live on rodents, enter the body via bites, abrasions or mucous membranes (65, 66) with cases normally occurring in urban areas (61, 67). Most cases present with a mild illness however, murine typhus can result in severe disease affecting the pulmonary, renal, gastrointestinal and neurological systems (68). Reporting of CNS infections in Vietnam due to *R. typhi* is however, rare. Gabor et al., 2022, found three cases of *Rickettsia* species in adults with clinically suspected CNS infections in Hanoi, which were co-infections with other pathogens (13) and in the study by Pommier et al., 2022, there was one case of *Rickettsia* species in a child in Hanoi who had encephalitis (14). This compares with 2.7% (*n* = 28) of cases of *Rickettsia typhi* among cases of suspected CNS infection in the study in Laos (63).

### *Leptospira* species

Leptospirosis is caused by the spirochaete *Leptospira* and is transmitted to humans via the urine of infected mammals, particularly rodents (69). Typically, those with leptospirosis experience fever, myalgia and headache however, in severe cases, renal failure, hepatic failure and pulmonary haemorrhagic may occur (69, 70). On occasion, there may be CNS manifestations (71, 72). Testing for leptospirosis can be challenging as culture and PCR both have a low sensitivity, the former due to the long incubation period required. The microscopic agglutination test is considered the gold standard diagnostic (73), which is technically challenging and often unavailable, likely resulting in under-diagnosis. Perhaps for these reasons, in recent studies of the aetiologies of CNS infections in Vietnam, either diagnostics were either not performed or no cases of leptospirosis were detected (11–15) (Supplementary Table S2). For comparison, *Leptospira* species were detected in the CSF 2.9% (*n* = 31) of patients with a suspected CNS infection in the study in Laos (63).

## Viruses

### Japanese encephalitis virus

Japanese encephalitis virus (JEV), an RNA *Flavivirus* of which there are five genotypes, is transmitted in an enzootic cycle between *Culex* mosquitoes, animals such as wading birds and pigs, and humans with the latter acting as dead-end hosts (74–82). The seroprevalence of JEV in pigs in Vietnam ranges from 60 to 100% depending on where the pigs were farmed and the age of the pig (83–85). Less than 1% of humans infected with JEV develop symptoms ranging from a mild flu-like illness to encephalitis (74, 86).

Japanese encephalitis (JE) has been a notifiable disease in Vietnam since 2017, based on the case definition for viral encephalitis of ‘fever greater than 38°C and a change in mental status, seizures, abnormal movements, tremor or spastic paralysis’. Case confirmation is based on the presence of anti-IgM antibodies in the CSF (87). While surveillance is largely passive, cases are also detected through sentinel site surveillance for meningoencephalitis in children aged up to 15 years. The incidence of JE is highest in the northern region of the country, where peaks are seen in the summer months of May–July. In the southern, central and highland regions, the incidence is relatively constant throughout the year (87, 88).

JE is seen most commonly in children. However, it may also be seen in adults as evidenced in studies in Vietnam (6, 9, 12). Cases in adults are more likely to be seen where there is no pre-existing immunity for example, in areas which have only recently experienced epidemics (87).

Vaccination against JE in Vietnam commenced in 1997, starting in high-risk provinces before expanding to all by 2014. Two doses of an inactivated mouse brain-derived vaccine are given to children aged 1–5 years, 1–2 weeks apart followed by a booster one year later (87). As a result of the vaccination programme, the national proportion of cases of AES which are due to JE has reduced (87, 89). However, the case incidence of JE still remains high in some provinces, particularly those in the northwest (90). As a result of this Vietnam conducts regular vaccination catch-up campaigns. However, it was recommended in the WHO 2006 position paper on Japanese Encephalitis Vaccines that the mouse brain-derived vaccines should gradually be replaced with the new generation JE vaccines which have a better safety profile (91). Japan is an example of the successful control of JE due to human and porcine vaccination, improved living conditions and vector control (92, 93). In Japan, more than 1,000 cases were reported per year prior to 1960 (94). However, since 2000, less than 10 cases have been reported per year with the exception of 2016 when 11 cases were reported (95).

### Dengue virus

Dengue virus (DENV) is a *Flavivirus* with all four serotypes found in Vietnam (96, 97). Transmission occurs primarily via the bite of an infected *Aedes aegypti* and less commonly an *Aedes albopictus* mosquito (98). In southern and central Vietnam, which report the majority of cases, transmission occurs throughout the year with a peak during the rainy season from July to September. Most cases in northern Vietnam occur in the city of Hanoi during the autumn months, with few cases seen during the winter. This is despite the ability of the mosquitoes to survive the cooler temperatures by residing in concrete tanks with broken lids (99). Dengue is a notifiable disease in Vietnam however, it is likely that there is significant under-reporting as many cases are not hospitalised (98). Approximately every 10 years there were large outbreaks including in 1987 (100, 101), 1998 (100, 102), 2009 (100, 103) 2017 (104). However, more recently, the time between outbreaks reduced with one occurring in 2022 (138). Some outbreaks have been reported to coincide with increased activity of the El Niño and La Niña weather patterns (103).

Of those infected with DENV who develop symptoms, most have a mild illness including headache, fever, myalgia and rash however, secondary infection with a heterologous serotype can increase the risk of severe dengue which can result in haemodynamic shock and multi-organ failure (96, 105). Some of those infected may develop CNS manifestations including meningitis, encephalitis or meningoencephalitis (106–108). DENV accounted for 2.0% (*n* = 50) of cases in adults and 1.5% (*n* = 30) of cases in children (Supplementary Tables S3, S4; Figure 1). In 2024, the Ministry of Health for Vietnam approved the tetravalent live attenuated Qdenga vaccine however, it is not part of the EPI (109).

### Enterovirus

Enteroviruses, belonging to the family *Picornavirdae*, are transmitted via the faecal-oral and respiratory routes, can cause a range of clinical manifestations from a mild flu-like illness to hand, foot and mouth disease (HFMD) with oral ulcers and a rash on the hands, feet and buttocks (89, 110) and meningitis or encephalitis if the virus crosses the blood–brain barrier (111, 112). The genotype enterovirus A71 (EV-A71) has a particular ability to result in severe neurological manifestations (112) however, it was not isolated in Vietnam until 2003 (89). Enteroviruses accounted for a slightly higher proportion of cases of CNS infections in children (3.6%, *n* = 71) compared to adults (2.0%, *n* = 50; Supplementary Tables S3, S4; Figure 1). Monovalent vaccines based on the EV-A71 sub-genotype C4 are licenced in China with vaccine effectiveness against HFMD caused by EV-A71 reported to be 63.4% for complete vaccination (113). However, these are not available in Vietnam.

### Herpes simplex virus

Herpes simplex virus (HSV) belongs to the family *Herpesviridae* (111). HSV type 1 (HSV-1) is transmitted via oral contact and HSV type 2 (HSV-2), via sexual contact. Most people who are infected with HSV-1 or HSV-2 have mild or no symptoms. However, in some, HSV type 1 (HSV-1) causes oral or genital blisters/ulcers (‘cold sores’) whereas HSV-2 causes genital blisters/ulcers (114). Herpes Simplex Encephalitis (HSE) is most commonly caused by the reactivation of latent herpes simplex virus type 1 (HSV-1). Meningitis, is however, more likely to be caused by herpes simplex virus type 2 (HSV-2) (111). HSE is the most common cause of viral encephalitis in adults in countries in Europe and North America (115, 116) however, vaccines against both HSV-1 and HSV-2 remain in development (117). Although it was also the most common viral aetiology among adults in Vietnam, the proportion of cases was still relatively low at 3.0% (*n* = 75; Supplementary Tables S3, S4; Figure 1).

## Parasites and fungi

Compared to countries in other parts of the world, particularly those in sub-Saharan Africa, the prevalence of HIV in Vietnam is low. The 2023 estimate of HIV among adults aged 15 to 49 years was 0.3% (118). Due to this, the proportion of cases of CNS infections caused by parasites and fungi which are most often associated with immunosuppression, is much lower than in areas with high HIV prevalence. However, data from 2020 estimated that in Vietnam, 3.1% of those who were newly diagnosed with acquired-immunodeficiency syndrome (AIDS) or were living with HIV and drug resistance to antiretroviral therapy, were positive for cryptococcal antigen and of these, 70% developed into cryptococcal meningitis (119). Additionally, of the studies in Supplementary Table S1, *Cryptococcus neoformans* accounted for 0.5% (*n* = 13) of cases of CNS infections among adults (Supplementary Tables S3, S4; Figure 1). *C. neoformans* is a yeast which is found in the environment including soil and avian guano. Those who are immunosuppressed, particularly those living with HIV who have a low CD4 count, are at risk of cryptococcosis, with the most common presentation being cryptococcal meningitis (120).

Similarly, malaria incidence in Vietnam is low. In 2022, there were 455 cases of malaria (approximately 0.47 cases per 100,000 population) with the country aiming to eliminate all species by 2030 (121). As a result of this, unlike in for example, sub-Saharan Africa, cases of CNS infection due to malaria are rarely reported in Vietnam. In the study of childhood encephalitis by Pommier et al., *Plasmodium falciparum* accounted for less than 1% of causes of CNS infections (14).

Although not reported in the studies in Supplementary Tables S1–S4, neurocysticercosis is a known parasitic cause of CNS infections in Vietnam however, diagnosis is based on magnetic resonance imaging (MRI). In a study of the safety and efficacy of praziquantel in the treatment of patients with neurocysticercosis in Hanoi between 2017 and 2020, 104 patients were diagnosed with the condition (122). Similarly, other parasitic causes of CNS infections which may have been missed in the studies may include *Angiostrongylus cantonensis* which accounted for 67.3% of 55 cases of eosinophilic meningitis between 2008 and 2014 in Ho Chi Minh City (123).

## Autoimmune

Autoimmune causes of encephalitis are associated with a variety of autoantibodies against the neuronal cell surface or synapse with the common being the anti-N-methyl-_D_-aspartic acid (anti-NDMA) receptor antibody and the anti-leucine rich, glioma-inactivated 1 [anti-LGI1; voltage-gated potassium channel (VGKC) antibody] (124). In a study conducted in the Hospital for Tropical Diseases, Ho Chi Minh City over a period of 18 months between 2015 and 2016, of the 99 patients admitted with encephalitis, nine tested positive for anti-NMDA receptor encephalitis (125). Two case reported of patients treated in Hanoi, have also been published (126) and, in a recent study of the aetiology of encephalitis in children in Hanoi, Pommier et al., reported six cases of anti-NDMA receptor encephalitis (14). A rare case of alpha-amino-3-hydroxy-5-methyl-4-isoxazole propionic acid (AMPA) receptor autoimmune encephalitis was also recently described in Ho Chi Minh City (127).

## Lychee toxicity

In both Vietnam and India, it was noticed that outbreaks of encephalitis coincided with the lychee harvesting season. A retrospective analysis conducted between of 239 paediatric patients with encephalitis from Bac Giang province, northern Vietnam, between 2004 and 2009, found an independent association between the surface proportion of litchi plantation and the incidence of acute encephalitis (128). A case–control study conducted in northern India in 2014, found that among 104 cases and the same number of controls, those who had eaten lychees were at higher odds of having an encephalopathy compared to those who had not (odds ratio 7.8 (95%CI 3.6-24). The encephalopathy was thought to be due to the effect of the hypoglycin A or methylenecyclopropylglycine (MCPG) toxins (129). Following this publication, it was reported that 247 cases of CNS disease occurring between 1960 and 2017 in China, had developed following the consumption of lychees (130) however, there have been no known further reports in Vietnam.

## Unknown aetiologies

In all studies of the aetiology of CNS infections in Vietnam, the cause continues to remain unknown in a large proportion of cases despite extensive diagnostics (11–15). This is not unique to Vietnam and, globally, unknown aetiologies account for a large proportion of CNS infections (131). However, those for which diagnostics are more challenging such as *Orientia tsutsugamushi*, or require imaging such as neurocysticercosis, may be under-reported, particularly the latter where the seroprevalence of *Taenia solium* is relatively high in at-risk populations (132). Other helminths such as *Angiostrongylus cantonensis* should be considered in those with a CSF eosinophilic pleocytosis and accounting for as many as 3% of cases of CNS infections (123, 133); and in returning travellers with gram negative meningitis, chronic infection with Strongyloides should be considered (134).

Following analysis of the spatio-temporal distribution of cases of acute encephalitis syndrome in Vietnam, which showed that the highest incidence was in the summer months in the northern provinces and a positive association with temperature, it was hypothesised that a number of cases were due to vector-borne diseases (135). Therefore, surveillance for vector-borne diseases, which are not necessarily routinely tested in patients with CNS infections, but for which Vietnam hosts competent vectors, such as chikungunya virus (CHIKV) and Zika virus (ZIKV) could be improved. Nipah virus (NiV), although never detected in human cases in Vietnam, may be considered in the differential diagnosis in any future outbreaks of encephalitis. NiV has been detected by reverse transcriptase polymerase chain reaction (RT-PCR) in urine samples of *Pteropus lylei* bats in Cambodia (136) and nearly half of the serum samples obtained from *Rousettus leschenaulti* bats in Hoa Binh province, Vietnam were positive for anti-NiV IgG by enzyme-linked immunosorbent assay (ELISA) (137).

## Conclusion

While the heterogeneity in testing strategies in research studies makes comparisons and trends difficult to assess and care should be taken in future studies employ comprehensive testing to provide comparable data, the majority of cases of CNS infections in Vietnam were caused by *S. suis* and JEV. We would therefore recommend continuing to provide risk communication messaging around the consumption and preparation of raw pig products as well as improving awareness of *S. suis* among healthcare workers, particularly where bacterial cultures may be negative due to the prior use of antibiotics. Surveillance for JE should be continued with catch-up vaccination campaigns where required, while vaccination should be considered for *S. pneumoniae* and *N. meningitis*. Despite the challenges in determining the unknown aetiologies of many CNS infections, we would recommend considering pathogens such as *Orientia tsutsugamushi* as well as arboviruses such as CHIKV or ZIKV in the differential diagnosis.
