# Supplementary material for: Review of the aetiologies of central nervous system infections in Vietnam
Source: Front Public Health. 2025 Jan 31;12:1396915. doi: 10.3389/fpubh.2024.1396915 (PMC11825750; doi:10.3389/fpubh.2024.1396915)
Supplement: Supplementary file 1 [file Data_Sheet_1.docx]

**Supplementary data**

**Table S1. Number and percentage of the aetiologies of CNS infections in each of the publications.**

| **Publication** | **Site(s)** | **Year(s) of recruitment** | **Case definition** | **Population** | **Number of**  **Cases** |
| --- | --- | --- | --- | --- | --- |
| Tran et al., 1998 (4) | HCMC | 1993-1996 | Meningitis with cloudy cerebrospinal fluid (CSF) or polymorphonuclear neutrophils above 50/µl and an albumin concentration in the CSF above 45g/l. | Children* | 86 |
| Nguyen et al., 2007 (5) | HCMC | 1996-2005 | Suspected bacterial meningitis | Adults^±^ | 435 |
| Tan et al., 2014 (6) | HCMC | 1996-2008 | CNS infections of presumed viral aetiology | Adults | 291 |
| Tan et al., 2010  (7) | HCMC | 2004 | Encephalitis | Children | 194 |
| Taylor et al., 2012 (8) | Hanoi | 2007-2008 | Suspected meningitis or encephalitis | Adults | 352 |
| Trung et al., 2012 (9) | Multiple | 2007-2010 | Clinically suspected CNS infection | Adults and children | 969 |
| An et al., 2014 (10) | Hanoi | 2012 | Viral encephalitis | Children | 565 |
| Ngo et al., 2021(11) | Hanoi | 2012-2014 | Suspected meningoencephalitis based on abnormal cerebrospinal fluid (CSF) results and with CSF available for testing by polymerase chain reaction (PCR) | Adults^‡^ | 408 |
| Brindle et al., 2022 (12) | Multiple | 2012-2016 | Clinically suspected CNS infection | Adults and children^¥^ | 933 |
| Gabor et al., 2022 (13) | Hanoi | 2014-2017 | Clinically suspected CNS infection | Adults^±^ | 137 |
| Pommier et al., 2022 (14) | Hanoi | 2014-2017 | Encephalitis | Children | 203 |
| Pallerla et al., 2022 (15) | Hanoi | 2019-2020 | Symptomatic meningitis | Adults^±^ | 30 |

* Aged between 30 days and 5 years

^±^ Defined as 15 years and over

^‡^ Defined as 16 years and over

^¥^ Adults defined as 18 years and over and children, younger than 18 years

**Table S2. Laboratory methods used to detect pathogens in each of the publications.**

| **Publication** | **CSF microscopy** | **CSF bacterial culture** | **CSF RT-PCR** | | | | | | | | | | | **CSF ELISA** | | **Other** |
| --- | --- | --- | --- | --- | --- | --- | --- | --- | --- | --- | --- | --- | --- | --- | --- | --- |
|  |  |  | Dengue virus | Enterovirus | Herpes simplex virus | *Streptococcus suis* | *Streptococcus pneumoniae* | *Haemophilus influenzae* | *Neisseria meningitidis* | *Mycobacterium tuberculosis* | *Rickettsia* species | *Leptospira* species | *Orientia tsutsugamushi* | anti-Japanese virus IgM | anti-dengue virus IgM |  |
| **Tran et al., 1998 (4)** | X (Gram stain) | X |  |  |  |  |  |  |  |  |  |  |  |  |  | X^±^ |
| **Nguyen et al., 2007 (5)** | X (Gram stain) | X |  |  |  |  |  |  |  |  |  |  |  |  |  |  |
| **Tan et al., 2014 (6)** |  |  |  | X | X | X | X | X | X |  |  |  |  | X | X | X^†^ |
| **Tan et al., 2010 (7)** |  |  | X^*^ | X | X | X | X | X | X |  |  |  |  | X | X | X^‡^ |
| **Taylor et al., 2012 (8)** | X (Gram stain, ZN stain, India ink stain in HIV positive patients) | X |  | X | X | X | X |  | X |  |  |  |  |  |  | Xˆ |
| **Trung et al., 2012 (9)** |  | X |  | X | X | X | X | X | X | X |  |  |  | X | X | X^¥^ |
| **An et al., 2014 (10)** |  |  |  |  |  |  |  |  |  |  |  |  |  |  |  | X^§^ |
| **Ngo et al., 2021 (11)** |  | X |  |  | X | X | X | X | X | X |  |  |  |  |  | X^¥ š^ |
| **Brindle et al., 2022 (12)** |  |  |  | X | X | X | X | X | X |  |  |  |  | X | X |  |
| **Gabor et al., 2022 (13)** | X | X | X |  | X | X | X | X | X | X | X | X | X |  |  | X^¥ ž^ |
| **Pommier et al., 2022 (14)** |  |  | X | X^***^ | X | X | X | X | X | X | X | X | X | X | X | X^#^ |
| **Pallerla et al., 2022 (15)** |  | X |  |  |  |  |  |  |  |  |  |  |  |  |  | X^¥ $^ |

^±^ CSF: Latex agglutination for *Haemophilus influenzae* type b (Hib), *Streptococcus pneumoniae*, *Neisseria meningitidis* type B and *Staphylococcus aureus*.

^†^ CSF: Real-time polymerase chain reaction (RT-PCR) for Epstein-Barr Virus (EBV), cytomegalovirus (CMV), varicella zoster virus (VZV), Nipah virus (NiV), influenza A and B viruses, generic flaviviruses, mumps virus; sequencing was conducted for specific flavivirus species. Serum: Enzyme-linked immunosorbent assay (ELISA) for rubella virus (EBV and anti-CMV IgM and IgG antibodies).

^*^ If serology and/or virus culture were suggestive of dengue.

^‡^ CSF: RT-PCR for influenza A virus, CMV, Me Tri virus/Semliki Forest virus, human parechoviruses and generic flaviviruses; virus isolation. Serum: Virus isolation.

^^^ CSF: biochemical tests for bacterial identification; mycobacterial culture if suspected tuberculous meningitis; PCR for VZV and if CSF negative and non-purulent meningitis, NiV; 16S rRNA in those with suspected bacterial meningitis.

^¥^ Blood culture

^§^ PCR and ELISA for unspecified pathogens (CSF and serum)

^š^ Eiken test for *Cryptococcus neoformans*

^ž^ MALDI-TOF VITEK® MS to detect bacterial colonies in blood and CSF: PCR for *Staphylococcus aureus*, *Pseudomonas aeruginosa*, *Klebsiella pneumoniae* and RT-PCR CMV, EBV, Parvovirus B19, measles virus, *Cryptococcus neoformans*, *Listeria monocytogenes*.

^**^ EV-A71

^#^ CSF: RT-PCR for *Streptococcus agalactiae*, *Bartonella* spp., *Brucella* spp., *Listeria monocytogenes*, *Plasmodium falciparum*, *Toxoplasma gondii*, JEV, mumps virus, human parechoviruses, adenovirus, chikungunya virus, flaviviruses, EBV, CMV, VZV, human herpes virus 6 and 7, measles virus, rabies virus, rubella virus, hepatitis A virus. Rapid diagnostic test (RDT): *Cryptococcus* spp antigen. Buffy coat: RT-PCR for *Leptospira* spp., *O. tsutsugamushi*, *Rickettsia* spp., DENV (1-4), *Bartonella* spp. NP/throat swab: RT-PCR for *Bordetella pertussis*, *Chlamydophila* *pneumoniae*, *H. influenzae* (including type B), *Klebsiella pneumoniae*, *Legionella* spp., *Moraxella catarrhalis*, *Mycoplasma pneumoniae*, *Salmonella* spp., *S. aureus*, *S. pneumoniae*, *Pneumocystis jirovecii*, adenovirus, bocavirus, human coronavirus 229E + NL63 + HKU1 + OC43, EV, CMV, NiV, influenza virus A, B and C, metapneumoviruses A/B, parainfluenza virus 1-4,human parechovirus 1-4, rhinovirus, respiratory syncytial virus A/B. Serum: RT-PCR for adenovirus, CHIKV, DENV 1-4, flaviviruses, HAV, HHV6, measles virus, mumps virus, rubella virus. Rapid Diagnostic Tests (RDT) for *Treponema pallidum* (IgM/G/A), DENV (1-4) (IgM, IgG and NS1). ELISA (IgM) for *O. tsutsugamushi*, *Rickettsia typhi*, DENV, JEV, hepatitis A virus. Whole blood: RT-PCR for *Bartonella* spp., *Brucella* spp., *Leptospira* spp., *O. tsutsugamushi*, *Rickettsia* spp., *P. falciparum*, DENV 1-4. RDT for *Plasmodium* spp. antigen.

^$^ 16S rNA sequencing with nanopore MinION

**Table S3. Percentage and 95% confidence intervals of the aetiologies of CNS infections in each of the publications.**

| **Publication** | **Adults/children** | **Aetiology** | **Percentage (95% confidence interval)** | **Number** |
| --- | --- | --- | --- | --- |
| Tran et al., 1998 (4) | Children | *Streptococcus suis*  *Streptococcus pneumoniae*  *Mycobacterium tuberculosis*  *Haemophilus influenzae*  *Neisseria meningitidis*  *Cryptococcus neoformans*  Japanese encephalitis virus  Dengue virus  Enterovirus  Herpes simplex virus  Other or dual pathogens  Aetiology unknown | 0  27.9 (19.0-38.8)  NA  34.9 (25.1-46.0)  0  0  NA  NA  NA NA  14 (7.7-23.5)  23.3 (15.1-33.8) | 0  24  NA  30  0  0  NA NA NA NA  12  20 |
| Nguyen et al., 2007 (5) | Adults | *Streptococcus suis*  *Streptococcus pneumoniae*  *Mycobacterium tuberculosis*  *Haemophilus influenzae*  *Neisseria meningitidis*  *Cryptococcus neoformans*  Japanese encephalitis virus  Dengue virus  Enterovirus  Herpes simplex virus  Other or dual pathogens  Aetiology unknown | 26.7 (22.6-31.1)  12.5 (9.7-16.2)  2.1 (1.0-4.0)  1.6 (0.7-3.4)  4.4 (2.7-6.9)  0.2 (0.1-1.5)  NA  NA  NA  NA  NA  NA | 116  55  9  7  19  1  NA  NA  NA NA  NA  NA |
| Tan et al., 2014 (6) | Adults | *Streptococcus suis*  *Streptococcus pneumoniae*  *Mycobacterium tuberculosis*  *Haemophilus influenzae*  *Neisseria meningitidis*  *Cryptococcus neoformans*  Japanese encephalitis virus  Dengue virus  Enterovirus  Herpes simplex virus  Other or dual pathogens  Aetiology unknown | 0  0  NA  0  0  NA  12.4 (8.9-16.8)  6.5 (4.1-10.2)  2.7 (1.3-5.6)  6.5 (4.1-10.2)  3.9 (2.0-6.9)  68.0% (62.3-73.2) | 0  0  NA  0  0  NA  36  19  19  8  11  198 |
| Tan et al., 2010 (7) | Children | *Streptococcus suis*  *Streptococcus pneumoniae*  *Mycobacterium tuberculosis*  *Haemophilus influenzae*  *Neisseria meningitidis*  *Cryptococcus neoformans*  Japanese encephalitis virus  Dengue virus  Enterovirus  Herpes simplex virus  Other or dual pathogens  Aetiology unknown | 0  3.1 (1.3-6.9)  NA  3.1 (1.3-6.9)  0  NA  25.8 (19.9-32.6)  4.6 (2.3-8.9)  9.3 (5.7-14.5)  0.5 (0.03-3.3)  8.8 (5.3-13.9)  44.9 (37.8-52.1) | 0  6  NA  6  0  NA  50  9  18  1  17  87 |
| Taylor et al., 2012 (8) | Adults | *Streptococcus suis*  *Streptococcus pneumoniae*  *Mycobacterium tuberculosis*  *Haemophilus influenzae*  *Neisseria meningitidis*  *Cryptococcus neoformans*  Japanese encephalitis virus  Dengue virus  Enterovirus  Herpes simplex virus  Other or dual pathogens  Aetiology unknown | 13.6 (10.3-17.8)  2 (0.9-4.2)  2.6 (1.3-5.0)  0  0.6 (0.1-2.3)  1.4 (0.5-3.5)  NA  NA  0.6 (0.1-2.3)  3.4 (1.9-6.0)  2.8 (1.5-5.3)  72.7 (68-77.5) | 48  7  9  0  2  5  NA  NA  2  12  10  257 |
| Trung et al., 2012 (9) | Adults | *Streptococcus suis*  *Streptococcus pneumoniae*  *Mycobacterium tuberculosis*  *Haemophilus influenzae*  *Neisseria meningitidis*  *Cryptococcus neoformans*  Japanese encephalitis virus  Dengue virus  Enterovirus  Herpes simplex virus  Other or dual pathogens  Aetiology unknown | 23. 8 (20.6-27.4)  5.7 (4.0-7.9)  5.5 (3.9-7.7)  0  0.6 (0.2-1.8)  0.3 (0.1-1.3)  1.8 (0.9-3.3)  3.7 (2.4-5.6)  3.2 (2.0-5.1)  3.6 (2.3-5.4)  3.9 (2.6-5.8)  47.8 (43.8-51.8) | 147  35  34  0  4  2  11  23  20  22  24  295 |
| Trung et al., 2012 (9) | Children | *Streptococcus suis*  *Streptococcus pneumoniae*  *Mycobacterium tuberculosis*  *Haemophilus influenzae*  *Neisseria meningitidis*  *Cryptococcus neoformans*  Japanese encephalitis virus  Dengue virus  Enterovirus  Herpes simplex virus  Other or dual pathogens  Aetiology unknown | 0  5.9 (4.3-8.2)  1.8 (0.9-3.2)  6.3 (4.5-8.5)  1 (0.4-2.2)  0  22.8 (19.6-26.3)  2.2 (1.3-3.8)  5.8 (4.1-8.0)  2.2 (1.3-3.8)  3.0 (1.9-4.8)  49.0 (45.1-53.0) | 0  37  11  39  6  0  142  14  36  14  19  306 |
| An et al., 2014* (10) | Children | *Streptococcus suis*  *Streptococcus pneumoniae*  *Mycobacterium tuberculosis*  *Haemophilus influenzae*  *Neisseria meningitidis*  *Cryptococcus neoformans*  Japanese encephalitis virus  Dengue virus  Enterovirus  Herpes simplex virus  Other or dual pathogens  Aetiology unknown | NA  NA  NA  NA  NA  NA  40.8  NA  14.3  31.9  NA  74.0 |  |
| Ngo et al., 2021 (11) | Adults | *Streptococcus suis*  *Streptococcus pneumoniae*  *Mycobacterium tuberculosis*  *Haemophilus influenzae*  *Neisseria meningitidis*  *Cryptococcus neoformans*  Japanese encephalitis virus  Dengue virus  Enterovirus  Herpes simplex virus  Other or dual pathogens  Aetiology unknown | 8.8 (6.3-12.1)  2.7 (1.4-4.9)  3.2 (1.8-5.5)  1.7 (0.8-3.7)  3.2 (1.8-5.5)  0.7 (0.2-2.3)  NA  0.2 (0.01-1.6)  NA  2.2 (1.1-4.3)  4.7 (2.9-7.3)  72.5 (67.9-76.8) | 36  11  13  7  13  3  NA  1  NA  9  19  296 |
| Brindle et al., 2022 (12) | Adults | *Streptococcus suis*  *Streptococcus pneumoniae*  *Mycobacterium tuberculosis*  *Haemophilus influenzae*  *Neisseria meningitidis*  *Cryptococcus neoformans*  Japanese encephalitis virus  Dengue virus  Enterovirus  Herpes simplex virus  Other or dual pathogens  Aetiology unknown | 16.2 (13.1-19.8)  4.6 (3.0-6.9)  NA  NA  0.6 (0.2-1.9)  0  2.2 (1.2-4.0)  1.4 (0.6-3.0)  1.6 (0.7-3.3) 1.8 (0.9-3.5)  3.0 (1.8-5.0)  68.6 (64.3-72.6) | 81  23  NA  NA  3  0  11  7  8  9  15  343 |
| Brindle et al., 2022 (12) | Children | *Streptococcus suis*  *Streptococcus pneumoniae*  *Mycobacterium tuberculosis*  *Haemophilus influenzae*  *Neisseria meningitidis*  *Cryptococcus neoformans*  Japanese encephalitis virus  Dengue virus  Enterovirus  Herpes simplex virus  Other or dual pathogens  Aetiology unknown | 2.1 (1.0-4.1)  6.1 (4.1-8.9)  NA NA  0.7 (0.2-2.2)  0  14.2 (11.1-18.0) 0.2 (0.1-1.5)  4.0 (2.4-6.4)  0.9 (0.3-2.5)  2.4 (1.3-4.3)  69.0 (64.3-73.3) | 9  26  NA NA  3  0  61  1  17  4  12  296 |
| Gabor et al., 2022 (13) | Adults | *Streptococcus suis*  *Streptococcus pneumoniae*  *Mycobacterium tuberculosis*  *Haemophilus influenzae*  *Neisseria meningitidis*  *Cryptococcus neoformans*  Japanese encephalitis virus  Dengue virus  Enterovirus  Herpes simplex virus  Other or dual pathogens  Aetiology unknown | 11.7 (7.0-18.6)  2.2 (0.6-6.8)  1.5 (0.3-5.7)  0  6.6 (3.2-12.5)  0.7 (0.04-4.6)  0  0  0  2.8 (0.9-7.8) 15.3 (10.0-22.7)  59.1 (50.4-67.3) | 16  3  2  0  9  1  0  0  0  4  21  81 |
| Pommier et al., 2022 (14) | Children | *Streptococcus suis*  *Streptococcus pneumoniae*  *Mycobacterium tuberculosis*  *Haemophilus influenzae*  *Neisseria meningitidis*  *Cryptococcus neoformans*  Japanese encephalitis virus  Dengue virus  Enterovirus  Herpes simplex virus  Other or dual pathogens  Aetiology unknown | 0.5 (0.03-3.1)  7.9 (4.7-12.7)  4.4 (2.2-8.5)  0.5 (0.03-3.1)  0.5 (0.03-3.1)  0  16.3 (11.6-22.2)  3.0 (1.2-6.6)  0  10.3 (6.7-15.6)  16.7 (12.0-22.8)  39.9 (33.2-47.0) | 1  16  9  1  1  0  33  6  0  21  34  81 |
| Pallerla et al., 2022 (15) | Adults | *Streptococcus suis*  *Streptococcus pneumoniae*  *Mycobacterium tuberculosis*  *Haemophilus influenzae*  *Neisseria meningitidis*  *Cryptococcus neoformans*  Japanese encephalitis virus  Dengue virus  Enterovirus  Herpes simplex virus  Other or dual pathogens  Aetiology unknown | 13.3 (4.4-31.6)  13.3 (4.4-31.6)  0  0  0  3.3 (0.2-19.1)  NA  NA  NA  NA  10.0 (2.6-27.7)  60.0 (40.8-76.8) | 4  4  0  0  0  1  NA  NA  NA  NA  3  18 |

* The numerators were not available for the study by Ngo et al., 2021 and therefore the 95% confidence intervals could not be calculated

**Table S4. Total number and percentage of cases (adults and children) across all twelve studies by aetiology.**

| **Pathogen** | **Number of cases** | **Percentage (95%CI)** |
| --- | --- | --- |
| **Adults (n=2542)** |  |  |
| *Streptococcus suis*  *Streptococcus pneumoniae*  *Mycobacterium tuberculosis*  *Haemophilus influenzae*  *Neisseria meningitidis*  *Cryptococcus neoformans*  Japanese encephalitis virus  Dengue virus  Enterovirus  Herpes simplex virus  Other or dual pathogens  Aetiology unknown | 448  138  67  14  50  13  58  50  38  75  103  1488 | 17.6 (16.2-19.2%)  5.4 (4.6-6.4)  2.6 (2.1-3.4)  0.6 (0.3-0.9)  2.0 (1.5-2.6)  0.5 (0.3-0.9)  2.3 (1.8-3.0)  2.0 (1.5-2.6)  1.5 (1.1-2.1)  3.0 (2.3-3.7)  4.0 (3.3-4.9)  58.5 (56.6-60.5) |
| **Children (n=1954)** |  |  |
| *Streptococcus suis*  *Streptococcus pneumoniae*  *Mycobacterium tuberculosis*  *Haemophilus influenzae*  *Neisseria meningitidis*  *Cryptococcus neoformans*  Japanese encephalitis virus  Dengue virus  Enterovirus  Herpes simplex virus  Other or dual pathogens  Aetiology unknown | 10  109  20  76  10  0  286  30  71  40  94  1208 | 0.5 (0.3-1.0)  5.6 (4.6-6.7)  1.0 (0.6-1.6)  3.9 (3.1-4.9)  0.5 (0.3-1.0)  0  14.6 (13.1-16.3)  1.5 (1.1-2.2)  3.6 (2.9-4.6)  2.0 (1.5-2.8)  4.8 (3.9-5.9)  61.8 (59.6-64.0) |
